# Suppression of adipocyte ABHD6 favors anti-inflammatory and adipogenic programs to preserve adipose tissue fitness in obesity

**DOI:** 10.1016/j.molmet.2025.102241

**Published:** 2025-08-29

**Authors:** Pegah Poursharifi, Camille Attané, Isabelle Chenier, Clemence Schmitt, Roxane Lussier, Anfal Al-Mass, Yat Hei Leung, Abel Oppong, Élizabeth Dumais, Nicolas Flamand, Mohamed Abu-Farha, Jehad Abubaker, Fahd Al-Mulla, Ying Bai, Dongwei Zhang, Marie-Line Peyot, André Tchernof, S.R. Murthy Madiraju, Marc Prentki

**Affiliations:** 1 Montreal Diabetes Research Center - Centre de Recherche du Centre Hospitalier de l'Université de Montréal, and departments of Nutrition and Biochemistry, University of Montreal, Montreal, Canada; 2 Québec Heart and Lung Institute Research Center, Université Laval, Québec City, Canada; 3 Translational Research Department, Dasman Diabetes Institute, Kuwait City, Kuwait; 4 Diabetes Research Centre, Beijing University of Chinese Medicine, Beijing, China

**Keywords:** α/β-hydrolase domain-containing 6, Adipose tissue, Insulin signaling, Obesity, Inflammation, Macrophages, Monoacylglycerol, PPARs

## Abstract

Some individuals exhibit metabolically healthy obesity, characterized by the expansion of white adipose tissue (WAT) without associated complications. The monoacylglycerol (MAG) hydrolase α/β-hydrolase domain-containing 6 (ABHD6) has been implicated in energy metabolism, with its global deletion conferring protection against obesity. However, the immunometabolic roles of adipocyte ABHD6 in WAT remodeling in response to nutri-stress and obesity are not known. Here, we demonstrate that in insulin resistant women, *ABHD6* mRNA expression is elevated in visceral fat and positively correlates with obesity and metabolic dysregulation. ABHD6 expression is also elevated in the WATs of diet-induced obese and *db/db* mice. Although adipocyte-specific ABHD6 knockout (AA-KO) mice become obese under high-fat diet, they show higher plasma adiponectin, reduced circulating insulin and inflammatory markers, improved insulin sensitivity, and lower plasma and liver triglycerides. They also show enhanced insulin action in various tissues, but normal glucose tolerance. In addition, AA-KO mice display healthier and less inflamed expansion of visceral fat, with smaller adipocytes and higher stimulated lipolysis and fatty acid oxidation levels. Similar but less prominent phenotype was found in the subcutaneous and brown fat depots. Thus, adipocyte ABHD6 suppression prevents most of the metabolic and inflammatory complications of obesity, but not obesity *per se*. Mechanistically, this beneficial process involves a rise in MAG levels in mature adipocytes, and their secretion, resulting in a crosstalk among adipocytes, preadipocytes and macrophages in the adipose microenvironment. Elevated intracellular MAG causes PPARs activation in adipocytes, and MAG secreted from adipocytes curtails the inflammatory polarization of macrophages and promotes preadipocyte differentiation. Hence, adipocyte ABHD6 and MAG hydrolysis contribute to unhealthy WAT remodeling and expansion in obesity, and its suppression represents a candidate strategy to uncouple obesity from many of its immunometabolic complications.

## Highlights

• ABHD6 expression in visceral fat correlates with metabolic dysregulation in obesity.
• Adipocyte ABHD6-KO mice show most of the traits of metabolically healthy obesity.
• Adipocyte secretion of MAGs promotes adipogenic and anti-inflammatory cues.
• ABHD6 contributes to unhealthy WAT remodeling and expansion in obesity.
• ABHD6 inhibition can be considered to improve adipose tissue health in obesity.

## Graphical abstract

**Figure:**
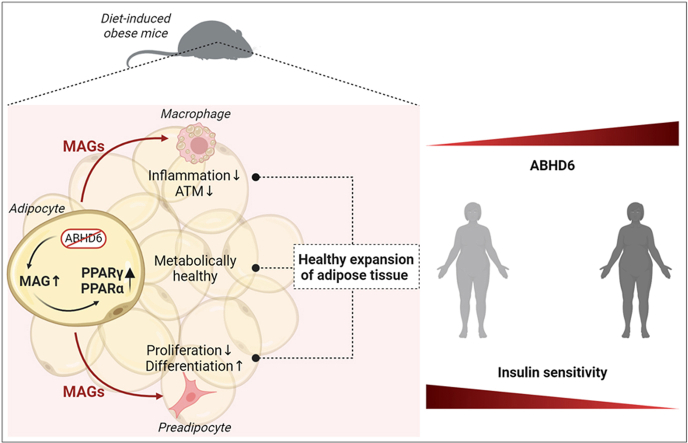

## 1 Introduction

White adipose tissue (WAT) is unique in its capacity to rapidly alter its size and cellular composition in response to nutritional and pathological cues [1]. The expansion of adipose tissue (AT) is not always associated with metabolic abnormalities [2,3]. In fact, AT insufficiency, as in lipodystrophy, or dysfunction as in obesity, results in ectopic fat deposition in other tissues, which is the hallmark of insulin resistance [4]. Expansion of AT during obesity through the recruitment of newly generated adipocytes (hyperplasia) is metabolically healthy, yet the enlargement of pre-existing fat cells due to fat accumulation (hypertrophy) causes metabolic complications [3]. Adipocyte inflammation, hypoxia, insulin resistance, apoptosis, and impaired lipid metabolism all contribute to unhealthy expansion of fat under energy surplus [3,5].

The concept of “metabolically healthy obesity” (MHO) has been proposed to be clinically important as a subset of people with obesity do not suffer from the detrimental effects of excess body fat [6]. MHO is generally associated with the expansion of subcutaneous fat, whereas excess visceral fat accumulation correlates with cardiometabolic disorders [7]. Despite multiple definitions, currently accepted features of MHO individuals include lower systemic and adipose inflammation, sustained adipogenesis and insulin sensitivity as well as reduced circulating and liver triglycerides (TG), in comparison to people who are obese and unhealthy and develop metabolic syndrome [[8], [9], [10]]. Also, rodent studies have suggested that among MHO characteristics, a salutary profile of AT function and metabolism is protective against the development of cardiometabolic complications [6]. Great progress has been made in understanding the mechanisms controlling obesity-mediated adipogenesis and AT insulin resistance and inflammation [1,11,12]. Previous work has demonstrated that adipose insulin signaling is closely linked to inflammatory pathways in response to obesity [[13], [14], [15]]. Notably, it was reported that obesity-induced insulin resistance in adipocytes is a key driver of AT macrophage recruitment [12]. These findings underscore the critical role of insulin signaling in AT and highlight the importance of cellular crosstalk within the adipose niche, as central mechanisms underlying obesity-induced complications. However, the molecular mediators that contribute to the preserved insulin sensitivity and healthy adipose expansion observed in MHO individuals are less clear.

Adipocytes store metabolic energy via lipogenesis in the form of TG within large cytoplasmic lipid droplets and liberate glycerol and free fatty acids (FFA) from TG via lipolysis in times of energy demand. The adipocyte TG pool is in a dynamic state, depending on the flux through the lipolysis and lipogenesis arms of the glycerolipid (GL) cycle, and the balance between these two pathways sets the fat mass [16,17]. The final exit step of the lipolytic segment of the GL cycle is catalyzed by two distinct monoacylglycerol (MAG) hydrolases: the traditional cytosolic MAG lipase (MAGL) and the relatively newly described membrane-bound α/β-hydrolase domain-containing 6 (ABHD6) [17,18]. We previously demonstrated that pan-deletion of ABHD6 in mice is protective against diet-induced obesity (DIO), insulin resistance, hepatic steatosis [19], and systemic and local AT inflammation [20]. Mechanistically, our earlier work has envisioned ABHD6 as a gatekeeper for the specific pools of signaling and intracellular MAG that activate peroxisome proliferator-activated receptors (PPARs) in various cells/tissues and thus contribute to the favorable phenotype of whole-body ABHD6-KO mice in the context of DIO [19,20]. Importantly, ABHD6 is expressed in many organs and regulates various biological processes [18]. For instance, ABHD6 in the brain contributes to the hydrolysis of endocannabinoids [21] that regulate body weight [22] and also governs neuroinflammation [23,24]. In macrophages, ABHD6 is a key component of pro-inflammatory responses to metabolic inflammation [20]. In pancreatic β-cell, ABHD6 is central to the regulation of glucose induced insulin secretion by hydrolyzing 1-MAG acting as a messenger coupling glucose metabolism to insulin secretion in β-cells [25,26]. Moreover, we have recently reported that adipocyte ABHD6 is a negative modulator of adaptive thermogenesis, as adipocyte-specific KO mice on a normal diet were resistant to hypothermia, an effect accounted for by enhanced thermogenic GL cycling in visceral fat [27]. Our earlier work has demonstrated that, in addition to MAGL which is highly expressed in WAT, ABHD6 also contributes substantially to MAG hydrolysis in adipocytes [19]. However, these two MAG lipases may play distinct immunometabolic roles. Notably, ABHD6 primarily localizes to the plasma membrane and nucleus or nuclear membrane in adipocytes, where it regulates signaling MAG-mediated transcriptional programs through PPARs [27]. In contrast, MAGL does not share this subcellular localization and has not been linked to adipose browning or thermogenesis, processes governed by PPARs activity. These observations underscore a unique and essential role for ABHD6 in adipocyte function. In fact, it remains unclear whether adipocyte ABHD6 plays a regulatory role in shaping the AT microenvironment and cellular network in response to obesity, particularly in the context of AT remodeling, expansion, and inflammation.

Here, we demonstrate that insulin resistance in women correlates with increased ABHD6 expression in visceral/omental WAT, and that adipocyte-specific ABHD6 deletion in mice facilitates healthy expansion and remodeling of WAT during obesity, without marked impact on weight gain. Our findings also provide mechanistic insights into the favorable effects of adipocyte ABHD6 suppression in obesity through cellular crosstalk within the adipose niche, particularly among mature adipocytes, preadipocytes and macrophages, and the activation of MAG/PPARs signaling, which promotes adipogenesis and attenuates inflammation.

## 2 Material and methods

### 2.1 Human study

#### 2.1.1 Subjects

Human WAT samples were obtained from 15 participants at Laval University Medical Center. Participants were all non-diabetic women with a mean age of 46.5 ± 3.7 years. Individuals were classified based on their homeostatic model assessment for insulin resistance (HOMA-IR) values (calculated from fasting glucose and insulin levels). HOMA-IR values bellow the 30th percentile was used as cut-off in defining the insulin sensitivity state.

#### 2.1.2 Individual characteristics and adipose sampling

Anthropometric measurements, including body weight, height, and sagittal diameter (using computed tomography), body fat distribution (by computed tomography at the L2L3 and L4L5 levels to assess total fat and subcutaneous and omental depot areas), and plasma lipid profiles were measured at the hospital and laboratories affiliated to Laval University, according to validated clinical procedures. Adipose tissue samples were collected from the subcutaneous and omental depots during gynecological surgery and were kept frozen until further analysis.

### 2.2 Animals

Mice were group-housed at room temperature (22 °C) on a 12 h light/dark cycle with ad libitum access to water and food. Age-matched adult male obese homozygous leptin receptor-deficient (*Lepr*^*db/db*^) mice and their lean control littermates (heterozygous *Lepr*^*db/+*^) were purchased from the Jackson Laboratories. *Abhd6*^*flox/flox*^ homozygous mice were generated as described before [25]. Homozygous *Abhd6*^*flox/flox*^ mice were bred with *Adipoq-Cre/ERT2* mice, both on C57BL6N background [28], to generate heterozygous *Abhd6*^*flox/+*^*/Adipoq-Cre/ERT2* mice, which were bred a second round with *Abhd6*^*flox/flox*^ mice to generate wild-type (WT), homozygous *Abhd6*^*flox/flox*^*/Adipoq-Cre* (used to obtain AT Adipose tissue-specific ABHD6 KO (AA-KO) mice by tamoxifen (TMX) injection), homozygous *Abhd6*^*flox/flox*^ (Fl/Fl) and *Adipoq-Cre* (Cre) mice. At 8 weeks of age, Fl/Fl, Cre and AA-KO male and female mice, fed chow diet, were given TMX (80 mg/kg body weight, dissolved in 10% ethanol in corn oil, oral gavage) every other day, for three times. After TMX treatment, mice were placed in single cages and were fed a high fat diet (HFD; Research Diets D12492; 60% calories from fat) for 12 weeks. To minimize bias, mice were randomly assigned to experimental groups using a stratified randomization approach based on initial body weight, ensuring comparable starting conditions. HFD has been shown to induce a robust formation of new adipocytes between 5 and 8 weeks of feeding [29]. Therefore, to ensure the complete deletion of ABHD6 in the fat depots upon HFD, we conducted a 2nd TMX treatment 7 weeks post HFD. Body weight and food intake were measured weekly. Body composition (lean and fat mass) was measured by magnetic resonance (EchoMRI Analyzer-700). The only exclusion condition was the development of dermatitis (1 Fl/Fl, 1 KO and 2 Cre). Experiments were conducted blindly until all data were collected and statistical analyses were completed. Mice were monitored daily and at the end of the experiments, animals were euthanized using an approved humane method in accordance with our institutional animal care guidelines.

### 2.3 *In vivo* metabolic studies

#### 2.3.1 Glucose tolerance test

Oral glucose tolerance test (OGTT) was conducted in 17 wk AA-KO, Cre, and Fl/Fl mice following 9 weeks on the HFD as indicated before [27]. After 6 h of food withdrawal, glucose (2 g/kg body weight) was administered orally. Blood was collected from the tail vein for glucose and insulin monitoring prior to gavage and at 15, 30, 60, 90, and 120 min after gavage.

#### 2.3.2 Insulin tolerance test

Intraperitoneal insulin tolerance test (ITT) was performed in 20 wk AA-KO, Cre, and Fl/Fl mice following 12 weeks on the HFD as indicated before [27]. After 4 h of food withdrawal, insulin (0.75 U/kg body weight, Humulin; Lilly, Indianapolis, IN, USA) was administered intraperitoneally and blood was collected from the tail vein for glucose monitoring prior to and at 15, 30, 45, 60, 90, and 120 min after injection.

#### 2.3.3 Analysis of insulin signaling

HFD-fed Fl/Fl and AA-KO mice (20 wk old) were fasted overnight, injected intraperitoneally with 1 U/kg insulin, and then sacrificed and tissues (gWAT, iWAT, iBAT, liver, and soleus muscle) were collected immediately. All tissues were harvested within 15 min after insulin injection and examined by immunoblot analysis for phospho-Akt (pSer473), Akt, and phosopho-IRβ (pTyr1146), and IRβ.

### 2.4 Cell culture and treatment

#### 2.4.1 Preadipocyte proliferation

3T3-L1 (American Type Culture Collection, Manassas, VA) were seeded in 6-well plates (5000 cells/well) and cell growth was monitored by counting the cells for four-six days before reaching confluency.

#### 2.4.2 3T3-L1 preadipocyte differentiation

3T3-L1 preadipocytes were differentiated two days post-confluency, with a differentiation cocktail containing 10 μg/ml (1.7 μM) insulin, 1 μM dexamethasone, and 500 μM 3-isobutyl-1-methylxanthine (IBMX). Two days later, the differentiation medium was replaced with DMEM medium supplemented with insulin only for 2 more days, and then changed to the regular medium [30]. 3T3-L1 adipocytes were fully differentiated by 8–10 days post induction. Adipocyte differentiation was monitored by microscopic evaluation of lipid droplet formation using Oil Red O staining.

#### 2.4.3 Mature adipocytes culture and conditioned-medium

Harvested fat pads (gWAT and iWAT) from mice were minced, washed, and then digested in collagenase type II (Sigma; 1 mg/mL in Krebs Ringer Buffer HEPES (KRBH) supplemented with 2% fatty acid–free BSA) for 45 min with shaking at 37 °C, followed by centrifugation at 1,500 rpm for 10 min. Floating mature adipocytes were collected, washed and then cultured for 24 h in serum-free DMEM with 0.5 % fatty acid–free BSA 100 U/ml penicillin and streptomycin. The conditioned-medium was harvested, centrifuged at 1000×*g* for 10 min at 4 °C to remove any cell debris, filtered through a 0.22 μm filter (Millipore), and kept at −80 °C for further analysis.

### 2.5 *Ex vivo* metabolic studies

#### 2.5.1 Lipolysis

Lipolysis was assessed by measuring glycerol and non-esterified fatty acid (NEFA) released from collagenase-isolated mature adipocytes or BAT explants under basal and stimulated (isoproterenol (ISO); 1 μM) conditions. Briefly, freshly isolated mature adipocytes (∼30,000/tube) were incubated in KRBH containing 2% fatty acid–free BSA and 5 mM glucose for 2 h at 37 °C with gentle shaking, and glycerol and NEFA release was normalized to total protein content. Commercial kits were used to measure glycerol (Sigma) and NEFA release (Wako diagnostic NEFA-HR).

#### 2.5.2 Fatty acid oxidation

Palmitate oxidation was measured in AT explants using [1–^14^C]-palmitate [19].

### 2.6 Lipidomics

Lipids were extracted as described previously with slight modifications [31]. In brief, samples were diluted to 0.5 ml with a Tris Buffer then mixed with 0.5 ml of LC/MS-grade MeOH containing 0.1 M AcOH and the deuterated internal standards. One milliliter of CHCl_3_ was then added to each sample, vortexed, and centrifuged at 3,000×*g* for 5 min. The CHCl_3_ fraction was harvested, and this step was repeated once for a total addition of 2 ml CHCl_3_. The organic phases were pooled and evaporated using a speed-vac evaporator and then suspended in 60 μl of mobile phase containing 50% of solvent A (water + 1 mM ammonium acetate + 0.05% acetic acid) and 50% of solvent B (acetonitrile/water (95/5; v/v) + 1 mM ammonium acetate + 0.05% acetic acid). 40 μl of each sample was finally injected onto an HPLC column (Kinetex C8, 150 × 2.1 mm, 2.6 μm; Phenomenex) and eluted at a flow rate of 400 μl/min using a discontinuous gradient of solvent A and solvent B (from 10% solvent A to 75% in 20 min, from 75% to 95% in 10 s, and held for 5 min at 95% re-equilibration to 10% solvent B in 3 min. Quantification of lipid mediators was carried out by HPLC system interfaced with a Shimadzu 8050 triple quadrupole mass spectrometer and using multiple reaction monitoring for the compounds and their deuterated homologs or a surrogate. Only peaks having the same retention time as the purified compounds of interest having and a signal-to-noise ratio greater than 5 were kept for quantification.

### 2.7 Tissue histology and analysis

Freshly dissected gWAT, iWAT, and iBAT from KO and Fl/Fl mice were fixed in 10% paraformaldehyde in PBS, dehydrated, embedded in paraffin, and sectioned. Sections were stained with hematoxylin and eosin (H&E) and stained for crown-like structures (CLS; activated macrophages) (anti-Mac-2 antibody from Biolegend). Images were processed for cell size and number quantification using ImageJ Version 1.48v (National Institute of Health, Bethesda, MD). Utilizing a semi-automated method [32], the average area, frequency distribution, and number of adipocytes in the gWAT was estimated. BAT lipid droplet size was also assessed. Briefly, images were converted to 8-bit grayscale, the threshold adjusted to mid grayscale range, converted to binary, segmented with watershed plugin, and finally the lipid droplet particles were analyzed automatically.

### 2.8 Immunoblotting

Tissues were extracted with ice-cold lysis buffer (20 mM Tris–HCl, pH 7.2, containing 150 mM NaCl, 1 mM EDTA, 1 mM EGTA, 1% (v/v) Triton X-100, 0.1% SDS, and protease inhibitors) and after protein quantification, 25–40 μg protein was used for Western blot analysis. For phosphorylation assays, membranes were first probed for phospho-proteins, then stripped using a re-blot strong antibody stripping solution (Sigma; 10-minute incubation), and subsequently re-probed for total proteins. Antibodies and dilutions are listed in Supplemental Table 2.

### 2.9 RNA extraction and qPCR

Total RNA was isolated from tissues and cells using the RNeasy Mini Kit, according to the manufacturer's instructions (Qiagen). Following reverse transcription of 2 μg RNA to cDNA, gene expression was determined by qPCR using SYBR Green. All gene expression analyses were run in duplicate and normalized to *18s* (mice) or *GAPDH* (human). Primer sequences are listed in Supplemental Table 3.

### 2.10 Plasma analysis

Glycerol, non-esterified fatty acids, triglycerides, and cholesterol were measured using commercially available kits. Circulating leptin was measured by Perkin Elmer AlphaLISA assay kit. Plasma adiponectin and insulin concentrations were assessed using mouse adiponectin and insulin ELISA kits. The rest of the cytokines were assessed by MILLIPLEX MAP Mouse Cytokine/Chemokine kit using the Luminex technology (Millipore).

### 2.11 Statistical analyses

Results are expressed as means ± SEM. All data were analyzed and graphs were prepared using GraphPad Prism 9 (GraphPad Software, San Diego, CA). Statistical differences between two groups were assessed by unpaired, two-tailed Student's *t* test, and between multiple groups using one-way or two-way analyses of variance (ANOVA), as indicated. Relationships between variables were assessed by linear regression analysis using Pearson correlation. A *p* value of less than 0.05 was considered statistically significant.

## 3 Results

### 3.1 ABHD6 expression in the visceral fat depots is positively correlated with the extent of obesity/insulin resistance/dyslipidemia in humans and in the *db/db* mice

In this study, we focus on the immunometabolic roles of adipose ABHD6 during obesity. Initially, we examined ABHD6 expression in the WAT depots of DIO and obese diabetic mouse (*db/db*) models. ABHD6 expression was markedly elevated in the gWAT from DIO and *db/db* mice (Figure 1 A, B), compared to the control mice, and a similar trend was observed in iWAT (Figure 1C, D). We further examined *ABHD6* mRNA expression in the visceral/omental and subcutaneous fat depots from a cohort of lean (body mass index (BMI) ≤ 24.99 kg/m^2^; n = 5) to overweight and severely obese (BMI ≥25 kg/m^2^; n = 10) female subjects and assessed *ABHD6* mRNA levels correlation with obesity/insulin resistance indices. Omental *ABHD6* mRNA levels positively and significantly associated with BMI, visceral tissue area at L2L3, sagittal diameter, homeostatic model assessment for insulin resistance (HOMA-IR), VLDL-cholesterol, and VLDL-TG (Figure 1E–K). There was a tendency for positive correlation between omental *ABHD6* mRNA and fasting insulin levels (r^2^ = 0.21, *p* = 0.07) (Figure 1L). When individuals were classified based on their HOMA-IR values, omental depot *ABHD6* level showed a significant increase in insulin resistance (IR) versus insulin sensitive (IS) groups (Figure 1M). Unlike the omental fat, *ABHD6* expression in the subcutaneous fat showed no significant difference between IR and IS subgroups (Figure 1N). No correlation was found between subcutaneous *ABHD6* and obesity and metabolic indices such as BMI and HOMA-IR (Figure 1 O, P). Overall, the results indicate a relationship between omental fat *ABHD6* expression and obesity-associated adipogenesis, insulin resistance and dyslipidemia.

**Figure 1:**
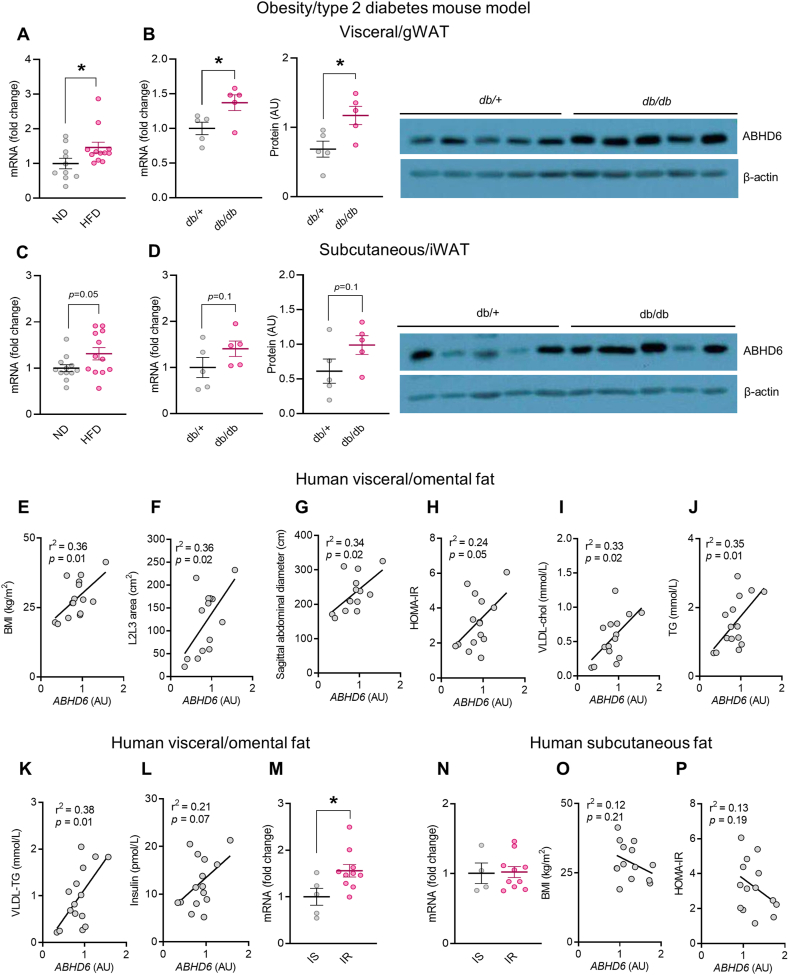
**ABHD6 expression in the visceral fat depots is positively correlated with the extent of obesity/insulin resistance/dyslipidemia in humans and in the *db/db* mice.A**- **D)** ABHD6 mRNA and protein expression in WAT from *db/+* control (glycemia prior to sacrifice: 6.9 ± 0.3 mM) and *db/db* mice (diabetic; glycemia prior to sacrifice: 31.3 ± 1.8 mM) (5 mice/group). Student's *t* test; ∗*p* < 0.05. **E-L, O and P)** Linear regression analysis (*r*^2^ and *p* values by Pearson correlation) of ABHD6 expression in the omental fat in female subjects vs. BMI (kg/m^2^), L2L3 area (cm^2^), sagittal abdominal diameter (cm), homeostatic model assessment for insulin resistance (HOMA-IR), VLDL-cholesterol (chol) (mmol/L), triglyceride (TG) (mmol/L), VLDL-TG (mmol/L) and insulin (pmol/L). **M** and **N)***ABHD6* mRNA levels.

### 3.2 High fat diet-fed adipocyte-specific ABHD6-KO mice display improved metabolic phenotype despite becoming obese

To further investigate the specific role of adipocyte ABHD6 in energy homeostasis, we employed an inducible adipocyte-specific ABHD6-KO (AA-KO) mouse model. We initially started the high-fat diet (HFD) regimen in both male and female mice. However, female mice displayed considerable variability in body weight, fat mass, and lean mass, with some showing resistance to HFD-induced weight gain. In addition, sex-associated differences in obesity-induced inflammation are well established in humans, with men exhibiting greater pro-inflammatory responses and women showing lower levels of the anti-inflammatory adipokine adiponectin [33]. Similarly, several rodent studies have reported that female mice are relatively protected from HFD-induced inflammation and insulin resistance in WAT [[34], [35], [36]]. In order to get statistically reliable data in female mice requires to markedly increase the number of mice per group, which is practically a difficult task as it involves several breeding cages to get the sufficient number of needed genotypes in females. Even if we do it, the results and conclusions are not likely change from what we saw with the male mice and the limited number of females. Because of these reasons, only male mice were used in the subsequent studies. We have previously validated the specific deletion of ABHD6 in the different AT depots of normal diet-fed AA-KO mice [27]. As it was shown before that HFD for 5–8 weeks induces new adipocyte generation [29], we ensured the complete deletion of ABHD6 in white and brown ATs by administering an additional dose of tamoxifen (TMX) during the 12 weeks period of HFD (Supplemental Figure 1A). As HFD-fed Cre and Fl/Fl mice displayed similar phenotype in terms of body weight, lean and fat mass, AT weight, oral glucose tolerance test (OGTT), and intraperitoneal insulin tolerance test (ITT) (Supplemental Figure 1 B-F), the rest of the experiments were carried out using Fl/Fl mice as the controls. In both male and female mice, there were no differences between HFD-fed AA-KO and Fl/Fl mice in their body weight, fat and lean mass, and weight of ATs (Figure 2 A-C, F–H). Also, plasma levels of glucose, glycerol, non-esterified fatty acids (NEFA) and cholesterol were similar between groups in male mice (Supplemental Table 1). Although glycemic and insulinemic responses to OGTT were similar between HFD-fed AA-KO and Fl/Fl mice in both sexes (Figure 2D,I), AA-KO mice exhibited improved insulin sensitivity, as seen in the ITT in males (Figure 2E) and to some extent in females (Figure 2J). No differences in glucose tolerance but improvement in insulin resistance could be due to the fact that a large amount of glucose disposal during OGTT is independent of insulin secretion or sensitivity [37]. In addition, differences in insulin clearance can directly influence the amount of insulin available to regulate blood glucose levels and, consequently, impact OGTT outcomes [38]. AA-KO mice also showed a significant decrease in fed insulinemia, and hepatic and plasma TG levels (Figure 2K–M). Plasma levels of the insulin-sensitizing adipokine adiponectin [39], and the granulocyte-colony stimulating factor (G-CSF), which protects the host from infection [40], were elevated in the AA-KO mice (Figure 2 N, O). Interestingly, despite its positive correlation with obesity, G-CSF has previously been shown to exert anti-obesity effects in a diabetic rat model [41]. In addition, the pro-inflammatory chemokine C-X-C motif ligand 9 (CXCL9) [42], IL-1α [43], and monocyte chemo-attractant protein 1 (MCP1) [44] were markedly reduced in the plasma from AA-KO *vs.* Fl/Fl mice (Figure 2O). Also, the obesity-associated chemokines (CXCL1 and CXCL10 [45]) and the pro-inflammatory cytokine IL-2 [46] showed a decreasing trend in the AA-KO *vs.* controls (Figure 2O).

**Figure 2:**
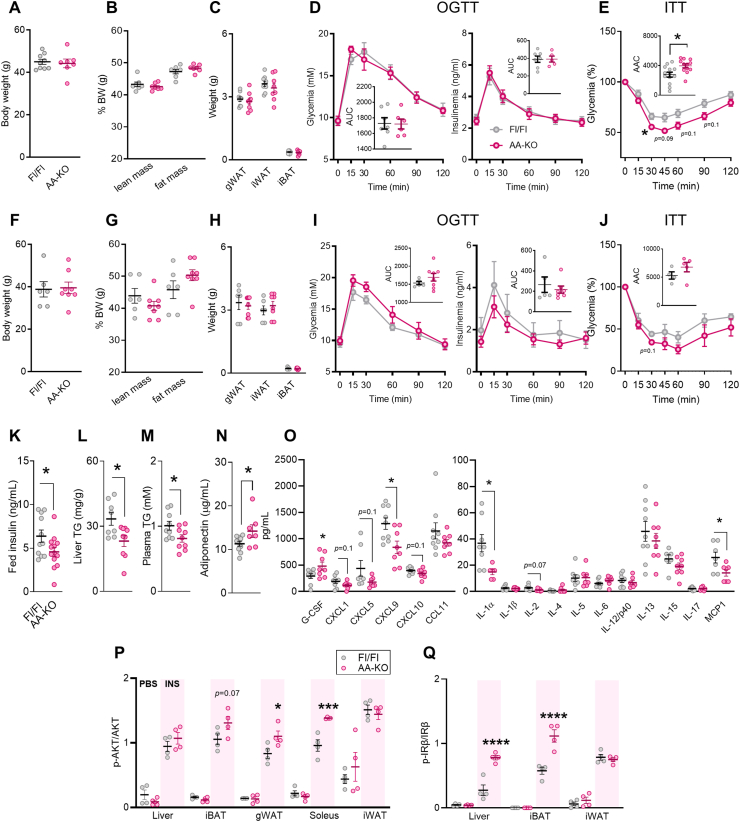
**High fat diet-fed adipocyte-specific ABHD6-KO mice display improved metabolic phenotype despite becoming obese.** Adipose tissue-specific ABHD6 KO (AA-KO) mice were generated as detailed in the Methods. After tamoxifen treatments, mice were fed a 60% a high fat diet (HFD) for 12 weeks (7–12 mice/group). A-E and K-Q male and F-J female mice. **A)** Body weight. **B)** Body composition by EcoMRI. **C)** Adipose tissues weight. **D)** Oral glucose tolerance test (OGTT); area under the curve (AUC). **E)** Insulin tolerance test (ITT); area above the curve (AAC). **F)** Body weight. **G)** Body composition by EcoMRI. **H)** Adipose tissues weight. **I)** OGTT. **J)** ITT. **K)** Fed insulin levels. **L)** Liver triglyceride (TG) content. **M)** Plasma TG. **N)** Plasma adiponectin. **O)** Plasma cytokines/chemokines. **P, Q)***In vivo* insulin signaling analysis. Student's *t* test (A, D, E, F-J); One-way ANOVA (B, C, K, L); Two-way ANOVA (D, E, I, J); ∗*p* < 0.05, ∗∗∗*p* < 0.001, ∗∗∗∗*p* < 0.0001.

*In vivo* Insulin signaling analysis demonstrated enhanced insulin-stimulated phosphorylation of AKT (pSer473) in the gonadal fat (gWAT) and soleus muscle, but not in the liver, interscapular brown AT (iBAT) or inguinal fat (iWAT) of HFD-fed AA-KO mice, compared with Fl/Fl controls (Figure 2P, Supplemental Figure 1 G-K). A significant increase in insulin-stimulated insulin receptor β (IRβ) phosphorylation (pTyr1146) was found in the liver and iBAT of HFD-fed AA-KO versus Fl/Fl mice (Figure 2Q, Supplemental Figure 1 I, J). The expression of phospho-IRβ was not clear in the gWAT and soleus muscle (not shown). Thus, despite similar weight gain as observed in controls, the HFD fed AA-KO mice were metabolically healthier, with improved insulin sensitivity/signaling, lower systemic inflammation and reduced basal insulinemia, plasma and liver TG and increased plasma adiponectin.

### 3.3 Adipocytes/lipid droplets are smaller in all fat depots from HFD-fed AA-KO mice

Histological examination by H&E staining showed that adipocytes/lipid droplets were smaller in gWAT, iWAT and iBAT of AA-KO compared to control mice (Figure 3A–C). In addition, the number of adipocytes was increased in gWAT and iWAT of AA-KO mice compared to Fl/Fl mice (Figure 3B). These data suggest a healthy expansion of AA-KO WAT, characterized by a higher proportion of smaller adipocytes (hyperplasia).

**Figure 3:**
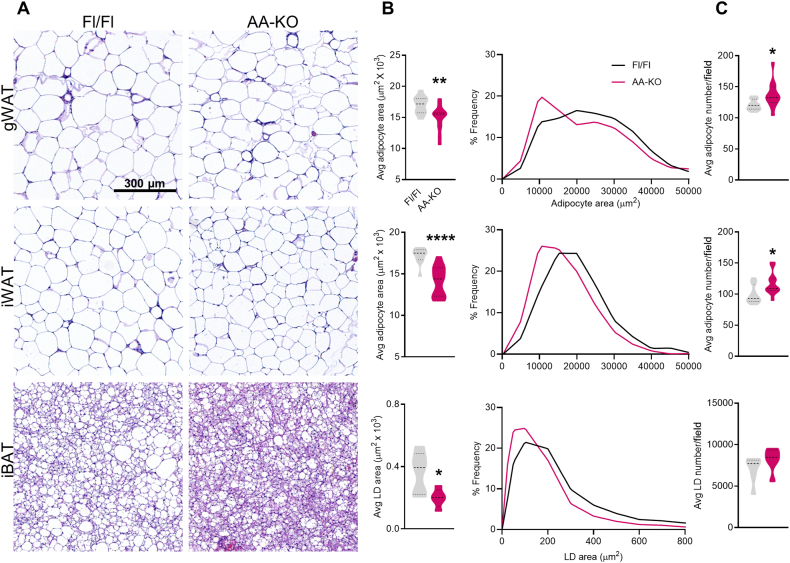
**Adipocytes/lipid droplets are smaller in all fat depots from HFD-fed AA-KO mice. Adipose tissues were harvested from HFD-fed mice.A)** Representative images from hematoxylin and eosin (H&E) stained sections of adipose depots (5 mice/group). Scale bar = 300 μm. **B)** Average adipocyte and lipid droplet (LD) area and number. **C)** The frequency distribution of adipocyte cell/LD size, expressed as percentage. Student's *t* test (B); ∗*p* < 0.05, ∗∗*p* < 0.01, ∗∗∗∗*p* < 0.0001.

### 3.4 Visceral fat from HFD-fed AA-KO mice exhibits enhanced stimulated-lipolysis and fat oxidation

**Figure 4:**
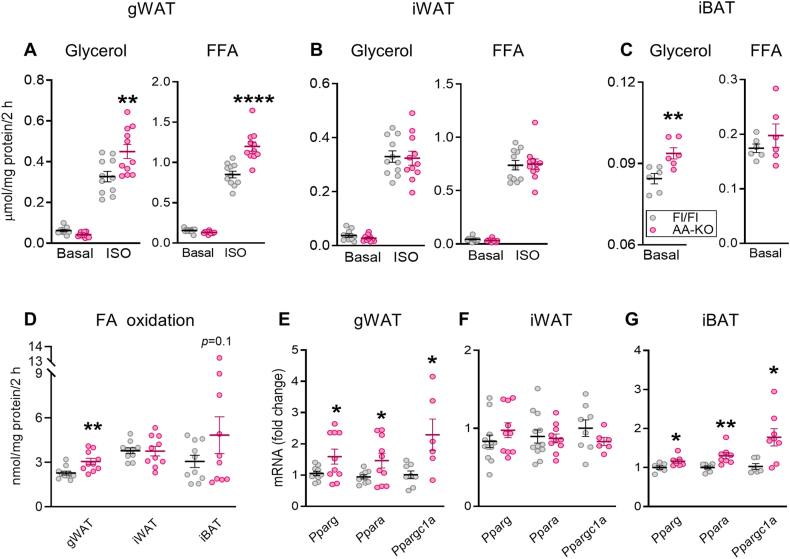
**Visceral fat from HFD-fed AA-KO mice exhibits enhanced stimulated-lipolysis and fat oxidation.** Adipose tissues were harvested from HFD-fed mice (n = 6–10 mice/group). **A-C)** Glycerol release from isolated mature white adipocytes and iBAT explants. **D-F)** Free fatty acid (FFA) release from isolated mature white adipocytes and iBAT explants. **G)** FA/palmitate oxidation. **H-J)***Pparg*, *Ppara* and *Ppargc1a* mRNA expression. Student's *t* test (C, F, G-J); One-way ANOVA (A, B, D, E); ∗*p* < 0.05, ∗∗*p* < 0.01, ∗∗∗∗*p* < 0.0001.

There were no differences between AA-KO and the control group in basal lipolysis, but the isoproterenol (ISO)-stimulated lipolysis measured as release of glycerol and NEFA were higher in the KO gWAT adipocytes (Figure 4A). Lipolysis in iWAT adipocytes were comparable between AA-KO and Fl/Fl mice (Figure 4B). However, iBAT explants from AA-KO mice showed increased basal glycerol release compared to the Fl/Fl (Figure 4C). Palmitate oxidation was elevated in the gWAT and exhibited an increasing trend in the iBAT (*p* = 0.1), without any changes in the iWAT from AA-KO mice (Figure 4D). Analysis of PPARs and related genes known to regulate lipolysis and FFA oxidation yielded results consistent with those of the lipid metabolic assays. Thus, HFD fed AA-KO mice exhibited upregulation of *Pparg*, *Ppara* and *Ppargc1a,* in the gWAT and iBAT but not in the iWAT (Figure 4E–G). Thus, adipose ABHD6 deletion prevents the obesity-associated adipocyte hypertrophy, and promotes the formation of smaller adipocytes that correspond to healthier AT [2]. In addition, the absence of adipocyte ABHD6 in gWAT enhances stimulated-lipolysis and fat oxidation and the expression of genes known to correspond to a better metabolic phenotype [47,48].

### 3.5 Adipocyte-specific ABHD6 deletion promotes anti-inflammatory profile of fat depots in obese mice

**Figure 5:**
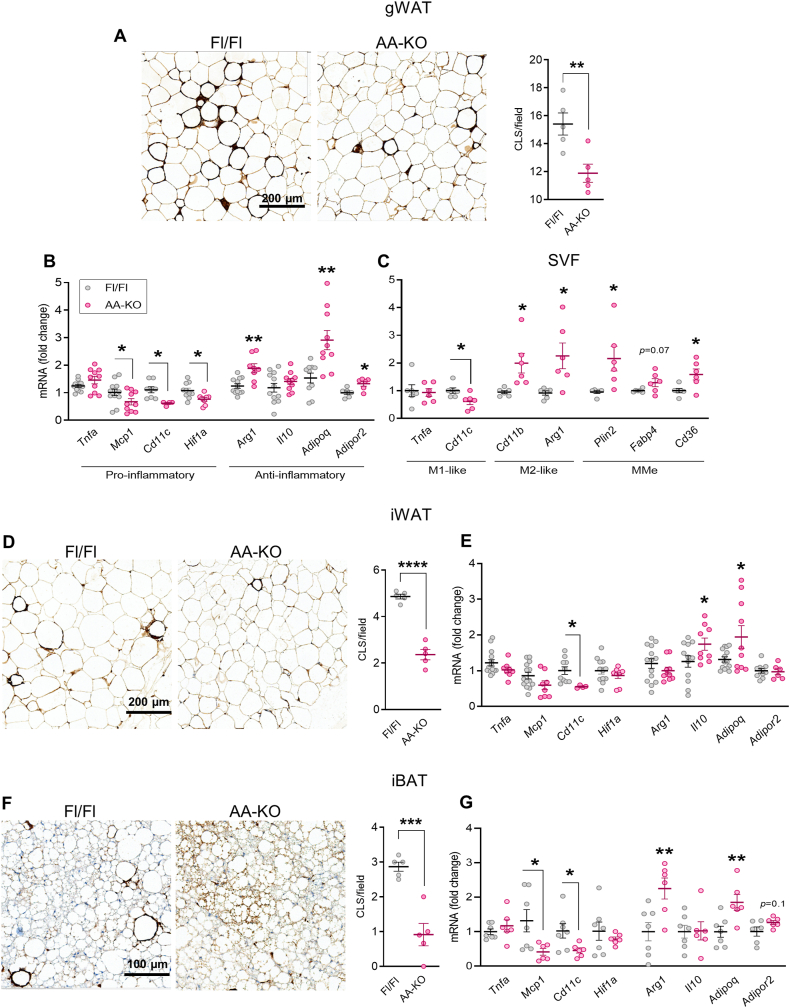
**Adipocyte-specific ABHD6 deletion promotes anti-inflammatory profile of fat depots in obese mice. Adipose tissues were harvested from HFD-fed mice.A, D, F)** Representative images from crown-like structures (CLS) stained sections of adipose depots. Quantification was performed as the average of 5 fields per depot per mouse, with data representing 5 mice per group. Scale bar = 200 μm. **B, C, E, G)** mRNA levels of the adipokines, cytokines, and pro/anti-inflammatory and macrophage markers (6–14 mice/group). Student's *t* test; ∗*p* < 0.05, ∗∗*p* < 0.01, ∗∗∗*p* < 0.001.

A hallmark of obesity associated insulin resistance is AT dysfunction due in part to increased macrophage infiltration and local inflammation [49,50]. We investigated the infiltration of macrophages by examining crown-like structures (CLS); inflammatory cells surrounding dead/dying adipocytes) in ATs by immunohistochemistry using anti-Mac2-antibody. The presence of CLS was markedly less in the gWAT from HFD-fed AA-KO mice compared to controls (Figure 5A). Consistent with the histological observations, mRNA levels of the pro-inflammatory markers *Mcp1, Cd11c* and hypoxia inducible factor 1α (*Hif1a*) were downregulated, while the anti-inflammatory markers arginase 1 (*Arg1*), adiponectin (*Adipoq)*, and adiponectin receptor 2 (*Adipor2*) were upregulated in the gWAT of HFD-fed AA-KO mice (Figure 5B). Similarly, the isolated stromal vascular fraction (SVF) from the AA-KO gWAT demonstrated decreased proinflammatory macrophage M1-like marker (*Cd11c*) and elevated anti-inflammatory macrophage M2-like (*Cd11b* and *Arg1*) and metabolically activated macrophage (MMe) markers (*Plin2* and *Cd36*) (Figure 5C). A comparable profile was also found in the iWAT and iBAT depots from the HFD-fed AA-KO mice (Figure 5D–G). Thus, the number of CLS and the expression of *Cd11c* was lower in the iWAT from AA-KO mice whereas there was an increase in the expression of *Il10* and *Adipoq* (Figure 5 D, E). Consistently, the amount of CLS and the mRNA levels of *Mcp1* and *Cd11c* were lower and *Arg1* and *Adipoq* were higher in the iBAT from AA-KO *vs.* controls (Figure 5 F, G). Together, these observations implicate a role for adipocyte ABHD6 in obesity-associated AT inflammation.

### 3.6 Conditioned-medium from ABHD6-deficient adipocytes promotes anti-inflammatory polarization of macrophages and preadipocyte differentiation

**Figure 6:**
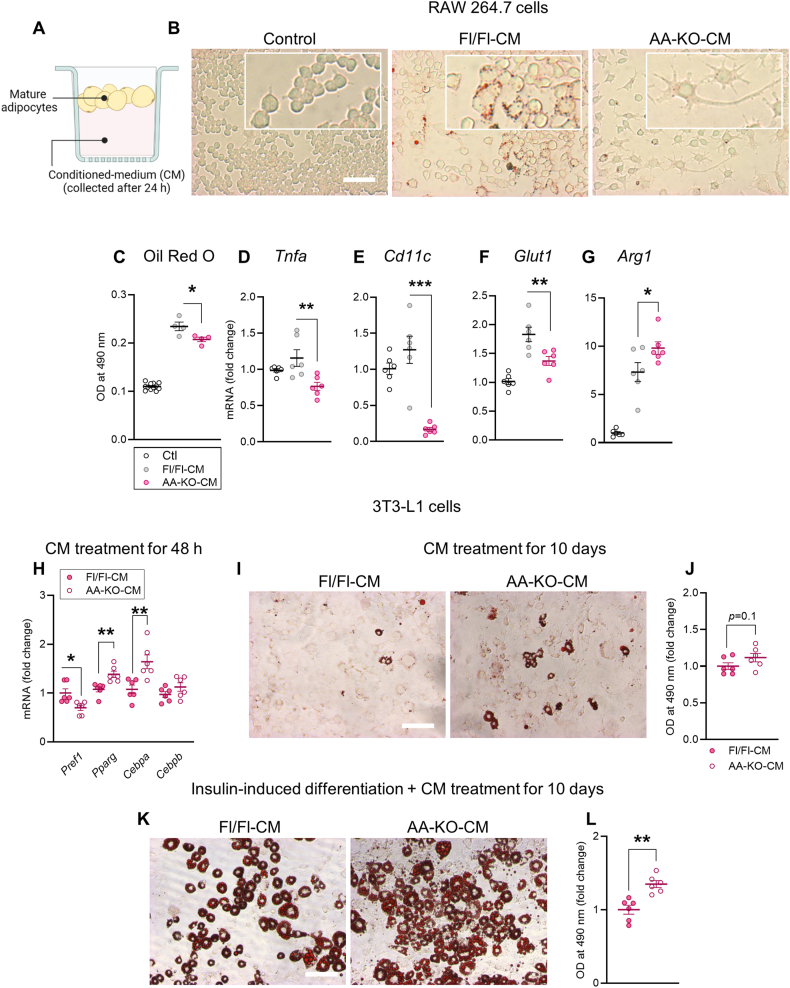
**Conditioned-medium from ABHD6-deficient adipocytes promotes anti-inflammatory polarization of macrophages and preadipocyte differentiation.** Conditioned-media (CM) were collected from gWAT mature adipocytes of HFD-fed AA-KO and Fl/Fl mice **(A)** and its inflammatory and pro-adipogenic effects were assessed in RAW 264.7 macrophages and 3T3-L1 cells, respectively (6 wells/condition). **B)** Representative images showing oil red O staining of RAW 264.7 macrophages before and after treatment with the CM. Scale bar = 100 μm. **C)** Oil red O quantification. **D-G)***Tnfa*, *Cd11c*, *Glut1* and *Arg1* mRNA levels in RAW 264.7 macrophages. **H)***Pref1*, *Pparg*, *Cebpa* and *Cebpb* mRNA levels in 3T3-L1 preadipocytes treated with CM for 48 h. **I)** Representative images showing oil red O staining of 3T3-L1 cells after 10 days of treatment with the CM. Scale bar = 100 μm**. J)** Oil red O quantification. **K)** Representative images showing oil red O staining of insulin-induced differentiated 3T3-L1 cells after 10 days of treatment with the CM. Scale bar = 100 μm**. L)** Oil red O quantification. One-way ANOVA (C–G); Student's *t* test (H, J, L); ∗*p* < 0.05, ∗∗*p* < 0.01, ∗∗∗*p* < 0.001.

Certain adipokines and lipids secreted from adipocytes have anti-inflammatory, insulin-sensitizing and pro-adipogenic effects [3]. As HFD did not induce adipocyte hypertrophy and inflammation in gWAT of AA-KO mice in which ABHD6 is deleted only in mature adipocytes, we hypothesized that ABHD6-ablated adipocytes secrete factors that maintain AT health and plasticity in the face of overnutrition. Conditioned-media (CM) were collected from gWAT mature adipocytes of HFD-fed AA-KO and Fl/Fl mice (Figure 6A) and tested for their effects on the phenotype and polarization of RAW 264.7 macrophages. As shown in Figure 6B, RAW 264.7 cells exposed to Fl/Fl-CM assumed a flattened, larger round shape appearance, while the AA-KO-CM-treated cells exhibited a higher degree of elongation, a characteristic feature of anti-inflammatory macrophages [51]. The latter cells also demonstrated less lipid accumulation (Figure 6C), markedly downregulated expression of pro-inflammatory markers *Tnfa*, *Cd11c* and *Glut1* (Figure 6D–F), and elevated expression of the anti-inflammatory marker *Arg1* (Figure 6G). Similar, but milder effects were observed when RAW 264.7 macrophages were treated with CM from AA-KO-iWAT and iBAT adipocytes (Supplemental Figure 2 A, B).

The pro-adipogenic property of CM from AA-KO and Fl/Fl adipocytes was examined using the 3T3-L1 cells. Treatment of confluent 3T3-L1 cells with AA-KO-CM versus Fl/Fl-CM for 48 h resulted in a marked increase in the mRNA levels of differentiation markers *Pparg* and *Cebpa,* along with a decrease in the expression of preadipocyte marker *Pref1* (Figure 6H). After 10 days of treatment with CM (added every two days), 3T3-L1 cells appeared to slightly differentiate into adipocytes, as demonstrated by Oil Red O staining and the appearance of few lipid droplets (Figure 6I). The quantification of Oil Red O images revealed a trend for increased lipid accumulation when cells were treated with AA-KO-CM compared to the Fl/Fl-CM (Figure 6J). Because of a very low degree of spontaneous differentiation with CM, we further induced adipocyte differentiation by insulin, and the CM was added during the differentiation process. Here, instead of the standard adipogenic cocktail (MDI) containing dexamethasone (DEX), 3-isobutyl-1-methylxanthine (IBMX) and insulin, which is commonly used for *in vitro* differentiation, we used a simplified approach with insulin alone to better replicate *in vivo* adipogenesis. Under this insulin-induced differentiation setting, 3T3-L1 cells treated with AA-KO-CM exhibited a higher degree of differentiation compared to Fl/Fl-CM (Figure 6 K, L). Thus, ABHD6-deleted mature adipocytes appear to endow protection against the detrimental effects of obesity in the AT microenvironment by secreting factor(s) that promote anti-inflammatory polarization of AT macrophages and enhance the differentiation capacity of preadipocytes.

### 3.7 Monoacylglycerol and adiponectin are increased in conditioned-medium of ABHD6-KO adipocytes and MAGs exert PPAR agonist like effects on the proliferation and differentiation of 3T3-L1 cells

**Figure 7:**
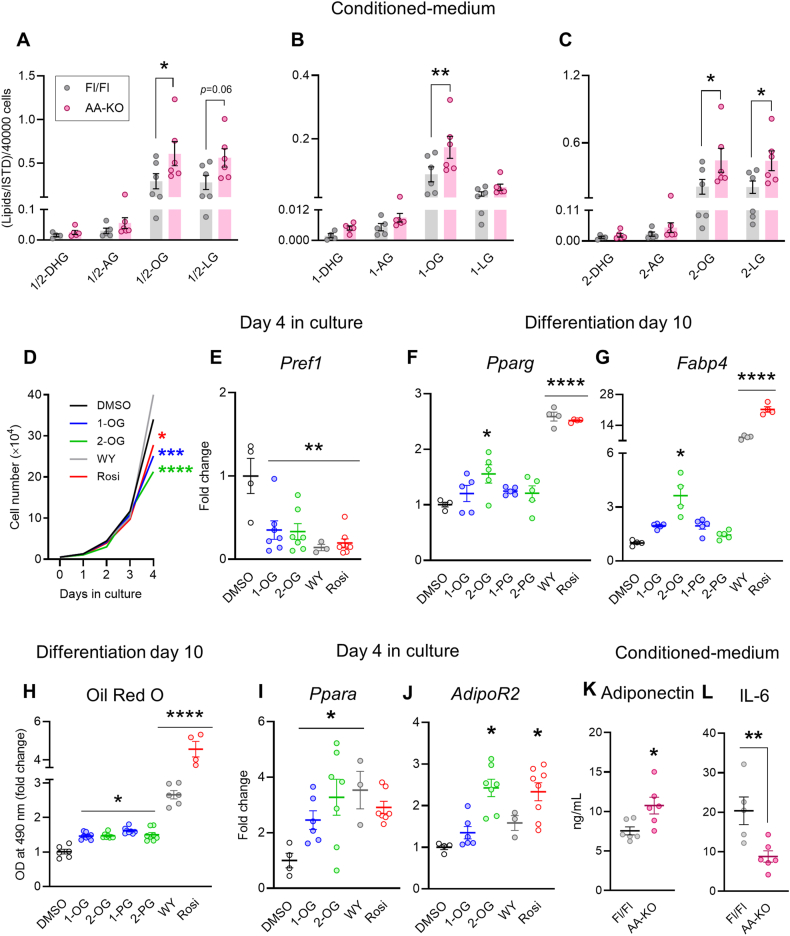
**Monoacylglycerol and adiponectin are increased in conditioned-medium of ABHD6-KO adipocytes and MAGs exert PPAR agonist like effects on the proliferation and differentiation of 3T3-L1 cells.A-C)** Analysis of 1/2-MAG (total MAG), 1-MAG, and 2-MAG species in the CM (6 mice/group). Depicted are analyte peak areas, normalized to respective internal standard (ISTD) peak areas, of representative lipids for each analyzed lipid class. **D)** Growth curve representing the number of 3T3-L1 preadipocytes of the indicated conditions at different time points (days 0, 1, 2, 3, and 4). Data points are the mean values of three independent experiments. **E)***Pref1* mRNA levels in 3T3-L1 preadipocytes. **F–H**) *Pparg*, and *Fabp4* mRNA levels and oil red O quantification in insulin-differentiated 3T3-L1 cells, following incubations with MAGs and PPAR agonists, as indicated. **I** and **J)***Ppara* and *AdipoR2* mRNA levels in 3T3-L1 preadipocytes following incubations with MAGs and PPAR agonists, as indicated. **K** and **L)** Adiponectin and IL-6 levels in the CM (6 mice/group). Total of 4–8 wells/condition for all the *in vitro* assays. Two-way ANOVA (A–D); One-way ANOVA (E–J); Student's *t* test (K, L); ∗*p* < 0.05, ∗∗*p* < 0.01, ∗∗∗∗*p* < 0.0001.

Our previous work demonstrated that ABHD6 acts as the main regulator of specific pools of signaling MAG that activates PPARs in the AT niche [19,20,27]. Indeed, here we have confirmed our earlier findings demonstrating higher levels of intracellular MAG species (oleoylglycerol (OG) and linoleoylglycerol (LG)) in isolated mature adipocytes from AA-KO versus Fl/Fl mice (Supplemental Figure 3 A-C). This prompted us to assess a novel MAG signaling mechanism in which ABHD6-deficient adipocytes with enhanced intracellular MAG levels secrete more MAG, contributing to the immunometabolic health of other AT-resident cells including ATMs and preadipocytes. Therefore, we further assessed endocannabinoid-related lipids, including the MAG species, in the CM with secreted lipids from mature adipocytes. Total OG, 1- and 2-OG, and 2-LG were significantly increased in the AA-KO CM versus control (Figure 7A–C). No marked difference was found between the two groups regarding the other lipid species, such as prostaglandins (PG), *N*-acylethanolamines (NAEs), and FAs (Supplemental Figure 3D). Interestingly, although the majority of oxylipins remained unchanged between two groups, AA-KO CM versus control contained higher levels of 13-hydroxy-octadecadienoyl glycerol (13-HODE-G), which is a MAG metabolite derived from 1/2-LG.

We then examined the potential beneficial effects of exogenous MAG on macrophage polarization and preadipocyte proliferation and differentiation. We have recently shown that the treatment of metabolically activated macrophages with exogenous 2-OG results in downregulation of pro-inflammatory markers and upregulation of anti-inflammatory markers, suggesting a favorable effect of MAG-mediated signaling in macrophage polarization in obesity [20]. We examined if exogenous MAG, in addition to its anti-inflammatory properties in macrophages [20], could also influence preadipocyte proliferation and/or differentiation. In pre-confluent 3T3-L1 cells, MAG species (both 1- and 2-OG) reduced 3T3-L1 proliferation, similar to the PPARγ agonist rosiglitazone (Rosi) (Figure 7D). Consistently, the expression of the preadipocyte marker *Pref1* [52] was reduced after 4 days of treatment with 1-OG, 2-OG or rosiglitazone and the PPARα agonists WY-14643 (WY) (Figure 7E). Pref-1 is known to maintain preadipocyte state and it is a negative regulator of adipocyte differentiation [53]. Accordingly, we further assessed the effect of exogenous MAGs on adipocyte differentiation capacity by treating the confluent 3T3-L1 cells with 1-OG, 2-OG, 1-palmitoylglycerol (1-PG), 2-PG or the PPARγ and PPARα agonists during the insulin-induced differentiation process. 2-OG caused a small increase in the mRNA expression of the differentiation and adipocyte markers *Pparg* and fatty acid-binding protein 4 (*Fabp4*) (Figure 7 F, G). In addition, Oil Red O data demonstrated positive effects of MAGs on adipocyte differentiation (Figure 7H). Because MAG-induction of adipocyte differentiation was less pronounced than with PPAR agonists and with the CM of the KO mice (Figure 6 K, L), we hypothesized that MAG alone may only partly contribute to the adipogenic effect of the AA-KO-CM, possibly by arresting the preadipocytes proliferation state. In addition, as the levels of *Ppara* and *AdipoR2* were enhanced in preadipocytes by MAGs, in particular upon 2-OG treatment (Figure 7 I, J), we further examined the levels of adipokines and other factors and metabolites in the CM. AA-KO-CM *vs.* control contained more insulin-sensitizing and pro-adipogenic adiponectin (Figure 7K) and also less pro-inflammatory IL-6 (Figure 7L). There were no changes in the levels of GM-CSF, CCL5, CCL11, IL-10, CXCL5, CXCL9, CXCL10, MCP1, glycerol, NEFA, TG and lactate between AA-KO-CM and the control CM (Supplemental Figure 3 E-H). Overall, deletion of ABHD6 in mature adipocytes resulted in enhanced secretion of MAGs and adiponectin in HFD-fed mice. Notably, while MAG species are the direct substrates of ABHD6, changes in adipokines such as IL-6 and adiponectin are likely indirect, arising from downstream activation of MAG/PPARs signaling.

## 4 Discussion

The link between obesity, insulin resistance and AT health and inflammation remains to be better understood [54,55]. Here, we have uncovered mechanisms underlying obesity-induced insulin resistance and unhealthy WAT expansion and inflammation by targeting adipocyte ABHD6. We show that deletion of ABHD6 specifically in mature adipocytes in adult mice mimics many features of MHO phenotype, except better glucose tolerance, as it markedly attenuates the HFD-induced metabolic and inflammation-related phenotypes but does not prevent the increase in body weight and fat mass. These *in vivo* findings were complemented by *ex vivo* and *in vitro* studies showing that the conditioned medium of adipocytes from gWAT of HFD-fed AA-KO mice (AA-KO-CM) markedly promotes adipogenesis in preadipocytes and inhibits pro-inflammatory polarization of macrophages.

Mechanistically, the anti-inflammatory effects of the AA-KO-CM can be at least in part due to the elevated levels of MAG in the KO mature adipocytes that is released in this medium, as we previously reported that exogenous 2-OG promotes the anti-inflammatory polarization of the metabolically activated macrophages [20]. We also earlier showed that exogenous 2-OG causes the activation of both PPARα and PPARγ [19] and similar dual PPAR activation was also seen in the macrophages by 2-OG and upon the suppression of ABHD6 either by its inhibitor KT203 or by ABHD6 deletion, both of which cause an increase in MAG levels [20]. In fact, dual agonism of PPARα and PPARγ pharmacologically was previously found to exert anti-inflammatory effects on macrophages [56,57]. Even though the dual-PPARα/γ agonists or “glitazars” were expected to show beneficial effects in metabolic diseases and obesity by a combination of insulin-sensitizing and lipid-lowering effects, their development was abandoned due to adverse cardiotoxic effects, which mainly arose because of a high level of PPARγ activation at the same time when PPARα is activated [58]. However, the unwanted cardiotoxic effect of a classical global PPARα/γ dual agonist, due to relatively higher activation of PPARγ [58], may not occur by ABHD6 inhibition mediated dual agonism of PPARα/γ as MAG generated by ABHD6 inhibition is a more potent trans-activator of PPARα than of PPARγ [19,27].

We also find that MAG promotes the commitment of preadipocytes towards adipogenic differentiation by retarding their proliferation possibly by downregulating Pref1, which is a prerequisite for the reduced proliferation of preadipocytes and adipogenic commitment [59]. It is noteworthy that as part of adipogenic commitment, preadipocytes undergo G1-cell cycle arrest and show reduced proliferation [60,61], as noticed in the present study by exogenous MAG treatment of the preadipocytes. How MAG in the AA-KO-CM can induce cell cycle arrest? Interestingly, dual agonism of both PPARα and PPARγ was shown to cause G1-cell cycle arrest in different cells [62]. Inasmuch as externally added 2-OG can act as an agonist of both PPARα and PPARγ in hepatocytes and 3T3-L1 preadipocytes [27], it is likely that MAG causes G1-cell cycle arrest of preadipocytes and then initiates adipogenic signaling. Thus, here we have identified distinct roles of adipocyte-secreted MAG acting as an anti-inflammatory factor in macrophages and as an inducer of adipogenesis in preadipocytes, both of which appear to be mediated via dual PPARα and PPARγ agonism.

Murine and human mature adipocytes interact with adipocyte progenitors/preadipocytes by releasing factors to regulate preadipocyte proliferation and/or adipogenesis [63,64]. Mature human adipocytes were shown to stimulate preadipocyte proliferation, and this effect was greater for adipocytes from obese subjects than those from lean individuals [65]. In the current study an interplay among three cell types, the ABHD6-deficient adipocytes, preadipocytes and macrophages was suggested to promote a MHO phenotype in HFD-fed AA-KO mice and to induce *de novo* adipogenesis, avert hepatic fat deposition, and prevent pro-inflammatory responses in obesity. Mechanistically, deletion of ABHD6 in mature adipocytes results in the accumulation of MAGs, which activate and upregulate PPARα and γ signaling [19,20,27], resulting in smaller fat cells, less inflammation and elevated stimulated lipolysis and fat oxidation. As expected, basal glycerol release in gWAT adipocytes from ABHD6-deficient mice showed a trend toward reduction; however, stimulated glycerol release was increased, likely due to the marked decrease in HFD-induced inflammation in this depot, as metabolic inflammation is known to suppress adrenergic-stimulated pathways [1,66]. Additionally, we find that these metabolically active fat cells release high levels of MAGs extracellularly, which could also exert dual PPARα/γ agonism in the neighboring preadipocytes, promoting their differentiation and in AT-resident macrophages, decreasing their pro-inflammatory responses. Notably, pharmacological activation of both PPARα and γ by a combination of Wy-14,643 and rosiglitazone has been shown to improve insulin sensitivity in the KKAy obese diabetic mouse model by enhancing circulating adiponectin level and the expression of its receptors in the WAT [67]. In fact, in addition to MAGs, AA-KO adipocytes release higher levels of adiponectin, which is a known adipogenic, insulin-sensitizing, and anti-inflammatory adipokine [68,69]. Considering that PPARγ is the key transcription factor controlling adiponectin expression [70], the activated MAG/PPAR signaling in the ABHD6-deleted adipocytes could stimulate adiponectin production and release. Therefore, it is likely that the elevated levels of adiponectin in AA-KO-CM contribute, at least in part, to the adipogenic and anti-inflammatory properties of adipocyte-derived CM from HFD-fed AA-KO mice. Adipocyte-derived IL-6, unlike that originating from immune cells, is known to promote HFD-induced AT inflammation in mice [71]. Thus, the lower levels of IL-6 in the AA-KO-CM could also be partly responsible for the observed lower degree of inflammation. These results collectively emphasize the importance of three molecules, i.e., elevated MAG and adiponectin, and decreased IL-6, that are released by the ABHD6-deficient adipocytes, in bestowing healthy metabolic and reduced inflammatory phenotype to HFD-fed AA-KO mice. It is noteworthy that HFD-fed whole-body MAGL-KO mice that show elevated MAG levels in different tissues were also found to exhibit better insulin sensitivity [[72], [73], [74]]. However, the molecular basis for the enhanced insulin sensitivity in the MAGL-KO mice is not known. The possible role of accumulating MAG in tissues in these whole-body MAGL-KO mice and if adipocyte MAGL plays any specific role in controlling insulin sensitivity was not investigated [[72], [73], [74]]. Thus, the possibility exists that accumulating MAG, because of the deletion of either ABHD6 or MAGL has beneficial effects, in alleviating insulin resistance, and our present study provides a plausible mechanism for this MAG-mediated protection.

In response to obesity, various fat depots expand differently. Hypertrophic gWAT is often linked with poor metabolic health, while iWAT is characterized as a healthier depot with high differentiation capacity [75]. These differences have been described by depot-specific microenvironments, cellular compositions and interconnections, signaling and developmental cues [76,77]. Indeed, individuals with MHO have lower amounts of ectopic fat (visceral and liver), but their subcutaneous fat shows higher capacity of expandability, that contributes to better insulin sensitivity compared to people with unhealthy obesity [7,10]. In fact, excess omental/visceral fat accumulation is the most commonly linked metabolic abnormality to insulin resistance seen in patients with abdominal/visceral obesity [78]. Interestingly, our present and the previous studies in mice [19,20,27] show that ABHD6 deletion leads to a more favorable metabolic profile in the gWAT, rather than iWAT depot. In line with this, we find a significant upregulation of ABHD6 mRNA and protein only in the gWAT, but not in iWAT of DIO and *db/db* mice. Notably, we report that in women who are clinically obese and overweight, ABHD6 expression in the omental/visceral fat depot is positively correlated with the extent of obesity and hyperlipidemia. Also, there was positive relationship between the increased omental/visceral ABHD6 expression and HOMA-IR and insulinemia in these individuals who are obese. Such correlations were not observed with the subcutaneous fat ABHD6 expression. Taken together, adipose ABHD6 plays distinct roles in different fat depots and in obesity its suppression remodels gWAT into a metabolically healthy fat depot.

The concept of MHO has often been challenged, with growing evidence suggesting that it may represent only a transient state that eventually progresses to unhealthy obesity over time [6,79,80]. Thus, it will be important to further investigate whether the favorable immunometabolic effects of adipocyte-specific ABHD6 deletion in obesity are sustained during prolonged HFD exposure and aging. Such studies could provide valuable insight into the potential of ABHD6 modulation to promote long-term adipose health and systemic metabolic resilience.

## 5 Conclusion

The results indicate that despite lacking a preventive effect on body weight gain, ABHD6 deletion in mature adipocytes under obesogenic diet conditions, establishes immunometabolic changes in the WAT niche, that favor healthier fat tissue expansion, insulin-sensitization in adipose, muscle and liver tissues, and reduced systemic inflammatory responses in mice, similar to many of the MHO features in humans. This is further strengthened by the observation that omental fat ABHD6 expression in human is directly correlated with obesity and metabolic dysregulation. Importantly, our results reveal that ABHD6 suppression in mature adipocytes institutes beneficial immunometabolic changes akin to those of a PPARα/γ dual agonist, without adverse effects. Overall, this study provides an insight into the pathways that underlie the healthy WAT expansion and remodeling upon adipocyte ABHD6 deletion that implicates three adipose cell types: the mature adipocytes, preadipocytes and macrophages. The emerging mechanism is that adipocyte ABHD6 deletion increases MAG levels in mature adipocyte and adipose MAG/PPARα/γ signaling and the increased MAG secreted from adipocytes drives *de novo* adipogenesis from pre-adipocytes and reduces WAT inflammation by promoting anti-inflammatory polarization macrophages. These findings provide support for ABHD6 as a therapeutic target for obesity-related complications, adipose health and insulin resistance.

## CRediT authorship contribution statement

**Pegah Poursharifi:** Writing – original draft, Visualization, Validation, Supervision, Methodology, Investigation, Formal analysis, Data curation, Conceptualization. **Camille Attané:** Visualization, Validation, Methodology, Investigation, Formal analysis. **Isabelle Chenier:** Methodology, Investigation, Data curation. **Clemence Schmitt:** Methodology, Investigation, Data curation. **Roxane Lussier:** Methodology, Data curation. **Anfal Al-Mass:** Methodology, Data curation. **Yat Hei Leung:** Methodology, Investigation, Data curation. **Abel Oppong:** Investigation, Data curation. **Élizabeth Dumais:** Methodology, Investigation, Formal analysis, Data curation. **Nicolas Flamand:** Writing – review & editing, Methodology, Investigation, Formal analysis, Data curation. **Mohamed Abu-Farha:** Writing – review & editing, Resources, Formal analysis, Data curation. **Jehad Abubaker:** Writing – review & editing, Resources, Formal analysis, Data curation. **Fahd Al-Mulla:** Writing – review & editing, Resources. **Ying Bai:** Methodology, Investigation, Data curation. **Dongwei Zhang:** Writing – review & editing. **Marie-Line Peyot:** Writing – review & editing, Validation, Supervision, Project administration, Methodology, Investigation, Conceptualization. **André Tchernof:** Writing – review & editing, Validation, Supervision, Investigation, Conceptualization. **S.R. Murthy Madiraju:** Writing – review & editing, Visualization, Validation, Supervision, Resources, Investigation, Funding acquisition, Conceptualization. **Marc Prentki:** Writing – review & editing, Visualization, Validation, Supervision, Resources, Investigation, Funding acquisition, Conceptualization.

## Statement of ethics

All the procedures for mouse studies were performed in accordance with the Institutional Committee for the Protection of Animals at the University of Montreal Hospital Research Center (CRCHUM).

For the human studies, the protocol was approved by the Research Ethics Committees of Laval University Medical Center (protocol number: 2014–2326)**,** and written informed consent was obtained from all the participants prior to their enrollment.

## Funding

This study was supported by funds from the Canadian Institutes of Health Research (#143308 to MP and SRMM) and Dasman Diabetes Research Institute/10.13039/501100003286Kuwait Foundation for the Advancement of Sciences (to MP, SRMM, MAF, JA, FAM). MP was recipient up to 2019 of a Canada Research Chair in Diabetes and Metabolism. PP was recipient of postdoctoral fellowships from the Fondation Valifonds, the Montreal Diabetes Research Center, the CRCHUM and Mitacs.

## Declaration of competing interest

The authors declare the following financial interests/personal relationships which may be considered as potential competing interests: Marc Prentki reports financial support was provided by Canadian Institutes of Health Research. If there are other authors, they declare that they have no known competing financial interests or personal relationships that could have appeared to influence the work reported in this paper.

## Figures and Tables

**figs1:**
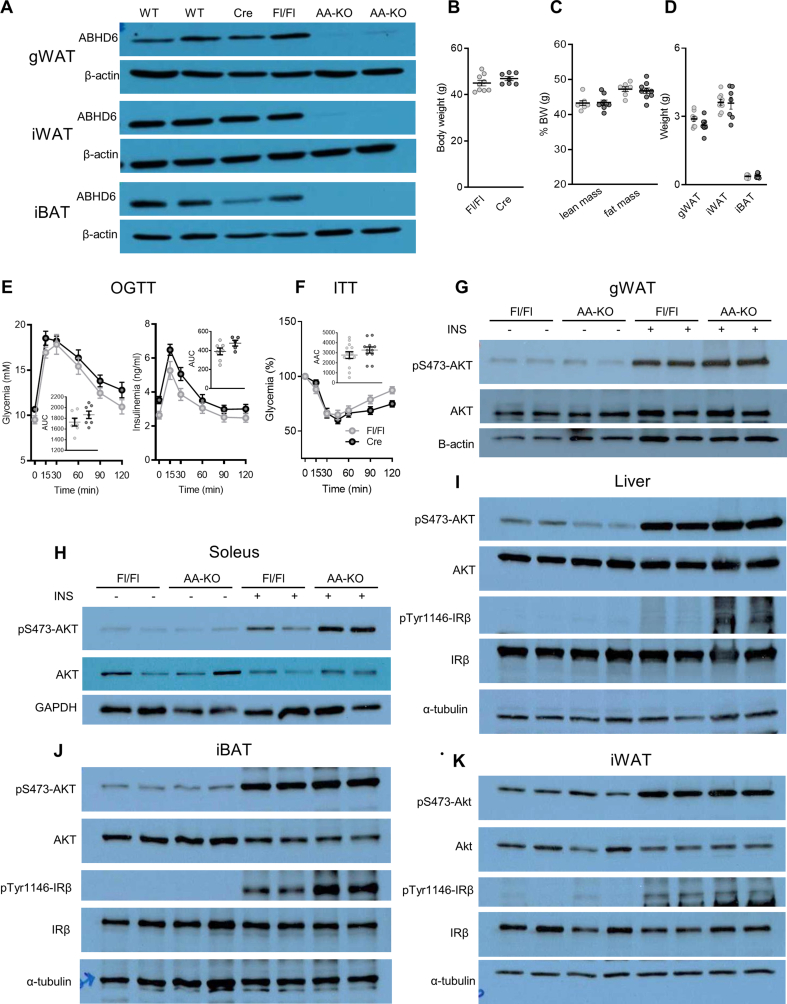


**figs2:**
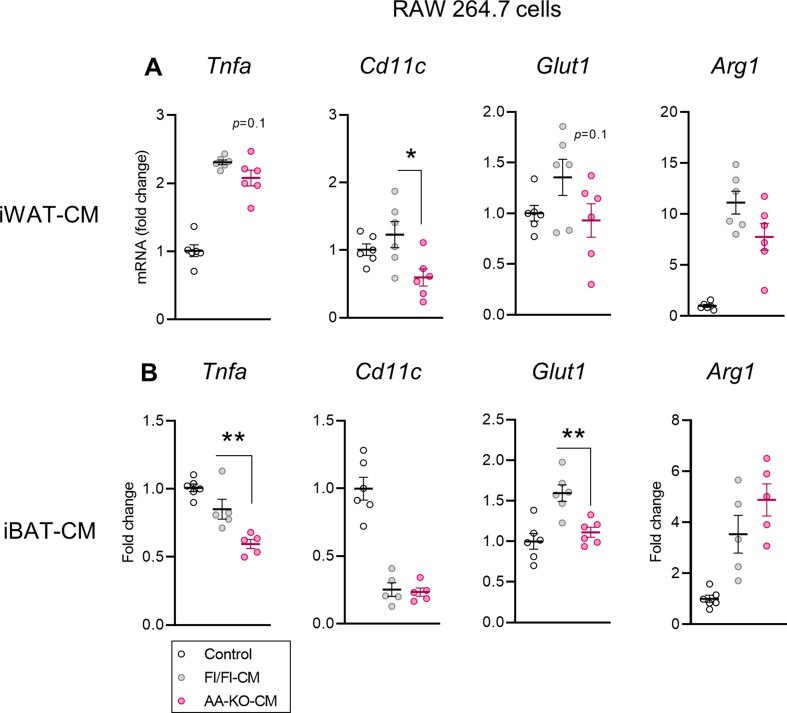


**figs3:**
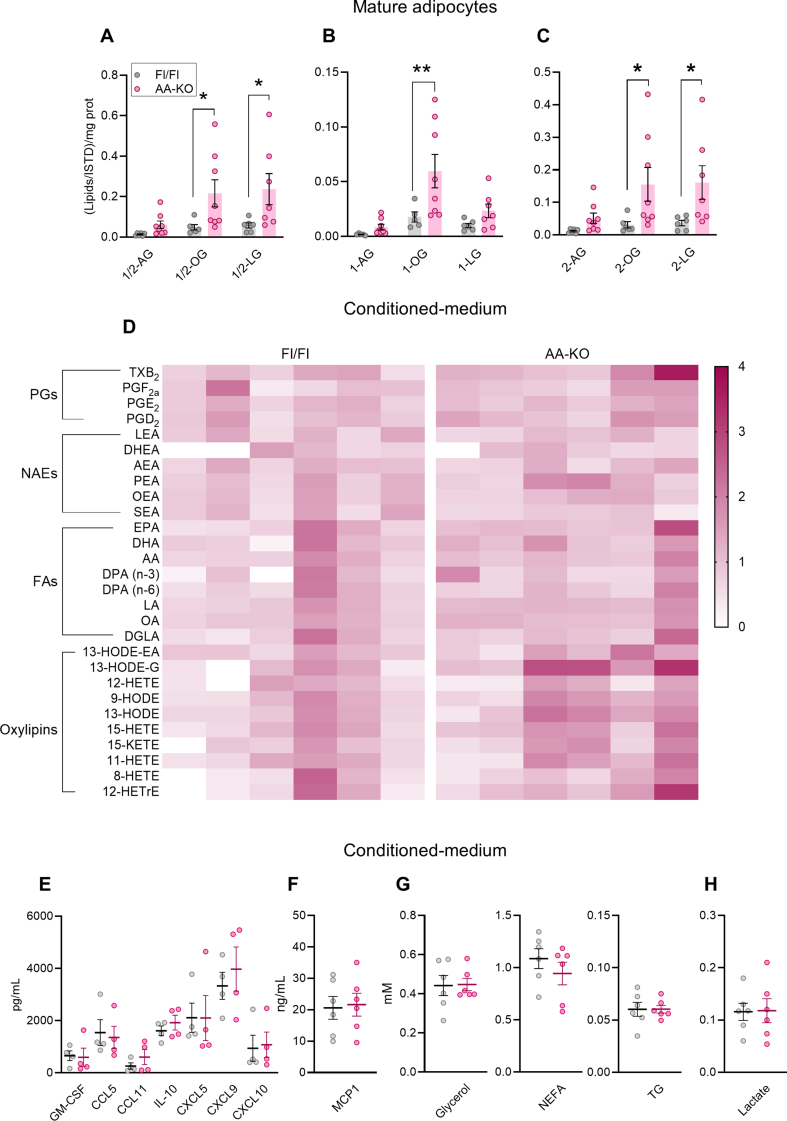


## Data Availability

Data will be made available on request.
