# Supplementary material for: Suppression of adipocyte ABHD6 favors anti-inflammatory and adipogenic programs to preserve adipose tissue fitness in obesity
Source: Mol Metab. 2025 Aug 29;101:102241. doi: 10.1016/j.molmet.2025.102241 (PMC12544160; doi:10.1016/j.molmet.2025.102241)
Supplement: Multimedia component 1 [file mmc1.docx]

**Supplemental file**

**Supplemental Figures**

**Figure S1. ABHD6 expression, body weight, fat mass, glucose and insulin tolerance and insulin signaling in ABHD6-KO and control mice.** After tamoxifen treatments, mice were fed a 60% a high fat diet (HFD) for 12 weeks (7-12 mice/group). **A)** ABHD6 protein expression. **B)** Body weight. **C)** Body composition by EcoMRI. **D)** Adipose tissues weight. **E)** Oral glucose tolerance test (OGTT). **F)** Insulin tolerance test (ITT). **G-K)** *In vivo* insulin signaling analysis. Mice were fasted overnight, injected intraperitoneally with 1 U/kg insulin, and then sacrificed and gWAT, iWAT, iBAT, liver, and soleus muscle were collected within 15 min after insulin injection and examined by immunoblot analysis for phospho-Akt (pSer473), Akt, and phosopho-IRβ (pTyr1146), and IRβ.

**Figure S2. Conditioned-medium from ABHD6-deficient iWAT and iBAT adipocytes promotes anti-inflammatory polarization of RAW 264.7 macrophages.**

Conditioned-medium (CM) was collected from iWAT and iBAT mature adipocytes of HFD-fed AA-KO and Fl/Fl mice and its effects on pro- and anti- inflammatory polarization was assessed in RAW 264.7 macrophages (6 wells/condition). **A** and **B)** *Tnfa*, *Cd11c*, *Glut1* and *Arg1* mRNA levels in RAW 264.7 macrophages. One-way ANOVA; **p* < 0.05, ***p* < 0.01.

**Figure S3. Increase in different monoacylglycerol species in ABHD6-KO adipocytes.**

**A-C)** Analysis of 1/2-MAG (total MAG), 1-MAG, and 2-MAG species in mature adipocytes (6-8 mice/group). Depicted are analyte peak areas, normalized to respective internal standard (ISTD) peak areas, of representative lipids for each analyzed lipid class. **D)** Heat map representation of lipids detected in conditioned-medium from ABHD6-KO (AA-KO) and Fl/Fl control adipocytes. Results are expressed as fold change in lipid metabolites in the AA-KO relative to the control group (6 mice/group). E-H) Cytokines and chemokines, glycerol, non-esterified fatty acids (NEFA), triglyceride (TG) and lactate levels in the CM (4-6 mice/group). Two-way ANOVA (A-C); Student's *t* test (E-H); **p* < 0.05, ***p* < 0.01.

**Supplemental Table 1**. Blood chemistry.

|  | **Fl/Fl** | **AT-KO** |
| --- | --- | --- |
| **Glycerol (mM)** | 0.47 ± 0.04 | 0.41 ± 0.04 |
| **FFA (mM)** | 0.63 ± 0.02 | 0.61 ± 0.04 |
| **Total Chol (mM)** | 2.54 ± 0.15 | 2.35 ± 0.11 |
| **Chol Esters (mM)** | 1.78 ± 0.13 | 1.72 ± 0.07 |
| **Free Chol (mM)** | 0.76 ± 0.10 | 0.63 ± 0.05 |
| **HDL (mg/dL)** | 104.64 ± 4.78 | 108.83 ± 4.88 |
| **LDL (mg/dL)** | 97.17 ± 4.88 | 94.88 ± 4.18 |
| **HDL/LDL** | 0.93 ± 0.03 | 0.91 ± 0.07 |
| **Fed glucose (mM)** | 8.12 ± 0.42 | 7.95 ± 0.25 |
| **Leptin (ng/mL)** | 220.77 ± 13.12 | 207.98 ± 20.92 |

**Supplemental Table 2**. **List of antibodies.**

| ANTIBODIES | SOURCE | REFERENCE |
| --- | --- | --- |
| Anti-ABHD6 | Cell signaling | 97573; clone: D3C8N |
| Phospho-Akt (Ser473) | Cell signaling | 4060; clone: D9E |
| Akt | Cell signaling | 9272 |
| Phospho-Insulin Receptor β (Tyr1146) | Cell signaling | 3021 |
| Insulin Receptor β | Cell signaling | 3025; clone: 4B8 |
| Mac-2 (Galectin-3) | BioLegend | 125402; clone: M3/38 |
| GAPDH | Cell signaling | 2118; clone: 14C10 |
| Anti-β-actin | Sigma-Aldrich | A5441; clone: AC-15 |
| Anti-α-tubulin | Abcam | ab4074 |

**Supplemental Table 3**. **Primer sequences used for RT-PCR.**

| Gene | | Primer Sequence |
| --- | --- | --- |
| *m18s* | Forward | CTG AGA AAC GGC TAG CAC ATC |
|  | Reverse | GGC CTC GAA AGA GTC CTG TAT |
| *mAbhd6* | Forward | AGA CCA GGT GCT TGA TGT |
|  | Reverse | CTC TCC ATC ACT ACC GAA T |
| *mFabp4* | Forward | CCA TCT AGG GTT ATG ATG CTC TTC |
|  | Reverse | ACA CCG AGA TTT CCT TCA AAC TG |
| *mPparg* | Forward | GGT CAG CTC TTG TGA ATG GAA |
|  | Reverse | ATC AGC TCT GTG GAC CTC TCC |
| *mPpara* | Forward | GGC CAT ACA CAA GGT CTC CAT |
|  | Reverse | AGA GAA TCC ACG AAG CCT ACC |
| *mPargc1α* | Forward | TAG AGT GTG CTG CTC TGG TTG |
|  | Reverse | GAT TGG TCG CTA CAC CAC TTC |
| *mPref1* | Forward | GAC CCA CCC TGT GAC CCC |
|  | Reverse | CAG GCA GCT CGT GCA CCC C |
| *mCebpa* | Forward | Ccg gga gaa ctc taa ctc |
|  | Reverse | Gat gta ggc gct gat gt |
| *mCebpb* | Forward | Gca aga gcc gcg aca ag |
|  | Reverse | Ggc tcg ggc agc tgc tt |
| *mAdipoq* | Forward | GTG TGT TCC TGC TTC ATT CC |
|  | Reverse | TGG TCA GAA GTT GGA GGT TC |
| *mAdipor2* | Forward | CCT TTC GGG CCT GTT TTA AGA |
|  | Reverse | GAG TGG CAG TAC ACC GTG TG |
| *mArg1* | Forward | AGA CCA CAG TCT GGC AGT TG |
|  | Reverse | CCA CCC AAA TGA CAC ATA GG |
| *mIl10* | Forward | CTT ACT GAC TGG CAT GAG GAT CA |
|  | Reverse | GCA GCT CTA GGA GCA TGT GG |
| *mTnfa* | Forward | CCA AGG CGC CAC ATC TCC CT |
|  | Reverse | GCT TTC TGT GCT CAT GGT GT |
| *mMcp1* | Forward | ATT GGG ATC ATC TTG CTG GT |
|  | Reverse | CCT GCT GTT CAC AGT TGC C |
| *mCd11c* | Forward | GTG CTG AGT TCG GAC ACA GT |
|  | Reverse | AGA GGC CAC CTA TTT GGT TAG T |
| *mHif1a* | Forward | GGG GAG GAC GAT GAA CAT CAA |
|  | Reverse | GGG TGG TTT CTT GTA CCC ACA |
| *mCd11b* | Forward | GGC TCC GGT AGC ATC AAC AA |
|  | Reverse | ATC TTG GGC TAG GGT TTC TCT |
| *mPlin2* | Forward | AAG AGC CAG GAG ACC ATT TC |
|  | Reverse | ACT CCA CCC ACG AGA CAT AGA |
| *mCd36* | Forward | AGG TCT ATC TAC GCT GTG TTC G |
|  | Reverse | CAA TGG TTG TCT GGA TTC TGG |
| *mGlut1* | Forward | Gct gtg ctt atg ggg ttc tc |
|  | Reverse | Cac ata cat ggg cac aaa gc |
| *hABHD6* | Forward | TGT GGT CAA GTT CCT TCC AAA |
|  | Reverse | TTG TTC AGC TTC AGG CAT TCT |
| *hGAPDH* | Forward | GAC CAC AGT CCA TGC CAT CAC |
|  | Reverse | AGG TCC ACC ACT GAC ACG TTG |
